# 2,2-Dimethyl-*N*-(phenyl­sulfon­yl)acetamide

**DOI:** 10.1107/S1600536809040483

**Published:** 2009-10-10

**Authors:** B. Thimme Gowda, Sabine Foro, P. G. Nirmala, Hartmut Fuess

**Affiliations:** a Department of Chemistry, Mangalore University, Mangalagangotri 574 199, Mangalore, India; b Institute of Materials Science, Darmstadt University of Technology, Petersenstrasse 23, D-64287 Darmstadt, Germany

## Abstract

In the title compound, C_10_H_13_NO_3_S, the N—H and C=O bonds in the SO_2_—NH—CO—C segment are *anti* to each other. The benzene ring and the SO_2_—NH—CO—C segment form a dihedral angle of 87.4 (1)°. The crystal packing features inversion-related dimers linked by pairs of N—H⋯O hydrogen bonds.

## Related literature

For sulfonamide drugs, see: Maren (1976 ▶). It has been postulated that the propensity for hydrogen bonding in the solid state can give rise to polymorphism due to the presence of various hydrogen-bond donors and acceptors, see: Yang & Guillory (1972 ▶). The hydrogen bonding preferences of sulfon­amides have also been investigated, see: Adsmond & Grant (2001 ▶). The nature and position of substituents play a significant role in the crystal structures of *N*-(ar­yl)sulfonoamides, see: Gowda *et al.* (2008*a* ▶,*b* ▶,*c* ▶);
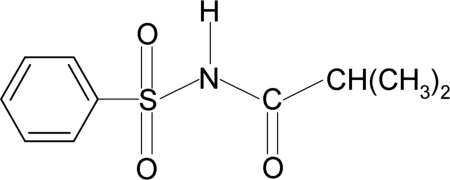


## Experimental

### 

#### Crystal data

C_10_H_13_NO_3_S
*M* *_r_* = 227.27
Monoclinic, 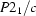

*a* = 6.1240 (4) Å
*b* = 22.201 (2) Å
*c* = 8.9192 (9) Å
β = 106.903 (6)°
*V* = 1160.26 (17) Å^3^
*Z* = 4
Cu *K*α radiation
μ = 2.40 mm^−1^
*T* = 299 K
0.50 × 0.13 × 0.08 mm

#### Data collection

Enraf–Nonius CAD-4 diffractometer
Absorption correction: none
2732 measured reflections
2075 independent reflections
1755 reflections with *I* > 2σ(*I*)
*R*_int_ = 0.047
3 standard reflections frequency: 120 min intensity decay: 1.0%

#### Refinement

*R*[*F* ^2^ > 2σ(*F* ^2^)] = 0.073
*wR*(*F* ^2^) = 0.213
*S* = 1.08
2075 reflections
140 parameters
H atoms treated by a mixture of independent and constrained refinement
Δρ_max_ = 0.78 e Å^−3^
Δρ_min_ = −0.58 e Å^−3^

### 

Data collection: *CAD-4-PC* (Enraf–Nonius, 1996 ▶); cell refinement: *CAD-4-PC*; data reduction: *REDU4* (Stoe & Cie, 1987 ▶); program(s) used to solve structure: *SHELXS97* (Sheldrick, 2008 ▶); program(s) used to refine structure: *SHELXL97* (Sheldrick, 2008 ▶); molecular graphics: *PLATON* (Spek, 2009 ▶); software used to prepare material for publication: *SHELXL97*.

## Supplementary Material

Crystal structure: contains datablocks I, global. DOI: 10.1107/S1600536809040483/fl2270sup1.cif

Structure factors: contains datablocks I. DOI: 10.1107/S1600536809040483/fl2270Isup2.hkl

Additional supplementary materials:  crystallographic information; 3D view; checkCIF report

## Figures and Tables

**Table 1:** Hydrogen-bond geometry (Å, °)

*D*—H⋯*A*	*D*—H	H⋯*A*	*D*⋯*A*	*D*—H⋯*A*
N1—H1*N*⋯O2^i^	0.81 (4)	2.12 (5)	2.898 (4)	160 (4)

Symmetry code: (i) 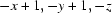.

**Fig. 1.**
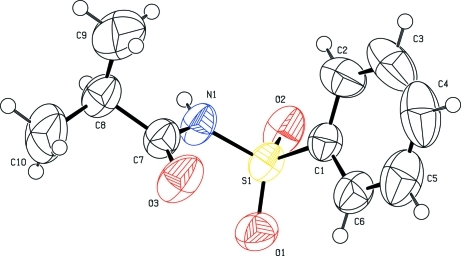
Molecular structure of (I), showing the atom labeling scheme. The displacement ellipsoids are drawn at the 50% probability level. The H atoms are represented as small spheres of arbitrary radii.

**Fig. 2.**
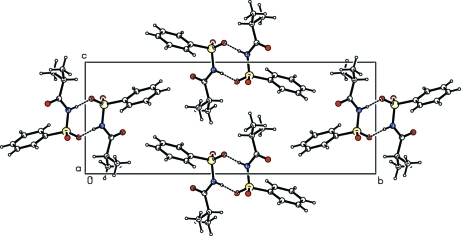
Molecular packing of (I) with hydrogen bonding shown as dashed lines.

## supplementary crystallographic information

### Comment

Sulfonamide drugs that exhibit antibacterial activity contain the sulfanilamide moiety (Maren, 1976). It has been postulated that the propensity for hydrogen bonding in the solid state, due to the presence of various hydrogen bond donors and acceptors, can give rise to polymorphism (Yang & Guillory, 1972). The hydrogen bonding preferences of sulfonamides have also been investigated (Adsmond & Grant, 2001). The nature and position of substituents play a significant role in the crystal structures of *N*-(aryl)sulfonoamides (Gowda *et al.*, 2008*a, b, c*).

As part of our substituent effect studies, the structure of (I) has been determined. The N—H and C=O bonds of the SO_2_—NH—CO—C segment in (I) are anti to each other (Fig. 1), similar to that observed in *N*-(phenylsulfonyl)2,2,2-trimethylacetamide (II)(Gowda *et al.*, 2008*c*), *N*-(phenylsulfonyl)2,2-dichloroacetamide (III) (Gowda *et al.*, 2008*a*) and other sulfonoamides (Gowda *et al.*, 2008*b*). The SO_2_—NH—CO—C segment forms a dihedral angle of 87.4 (1)° with the benzene ring, compared to values of 83.2 (1) and 76.0 (1)° (for the two independent molecules of (II)) and 79.8 (1)° in (III). In the crystal the molecules form inversion-related dimers along the *c* axis, linked by pairs of N—H···O(S) hydrogen bonds (Table 1, Fig.2).

### Experimental

The title compound was prepared by refluxing benzenesulfonamide (0.10 mole) with an excess of isobutanoyl chloride (0.20 mole) for about an hour on a water bath. The reaction mixture was cooled and poured into ice cold water. The resulting solid was separated, washed thoroughly with water and dissolved in warm dilute sodium hydrogen carbonate solution. The title compound was reprecipitated by acidifying the filtered solution with glacial acetic acid. It was filtered, dried and recrystallized from ethanol. The purity of the compound was checked by determining its melting point. It was characterized by recording its infrared spectra. Single crystals of the title compound used for X-ray diffraction studies were obtained from a slow evaporation of an ethanolic solution of the compound.

### Refinement

The H atom of the NH group was located in a difference map and and its position refined with N—H = 0.81 (4) Å. The other H atoms were positioned with idealized geometry using a riding model with C—H = 0.93–0.98 Å.

All H atoms were refined with isotropic displacement parameters (set to 1.2 times of the *U*_eq_ of the parent atom).

### Crystal data

**Table:**

C_10_H_13_NO_3_S	*F*(000) = 480
*M**_r_* = 227.27	*D*_x_ = 1.301 Mg m^−^^3^
Monoclinic, *P*2_1_/*c*	Cu *K*α radiation, λ = 1.54180 Å
Hall symbol: -P 2ybc	Cell parameters from 25 reflections
*a* = 6.1240 (4) Å	θ = 4.0–20.3°
*b* = 22.201 (2) Å	µ = 2.40 mm^−^^1^
*c* = 8.9192 (9) Å	*T* = 299 K
β = 106.903 (6)°	Rod, colourless
*V* = 1160.26 (17) Å^3^	0.50 × 0.13 × 0.08 mm
*Z* = 4	

### Data collection

**Table:**

Enraf–Nonius CAD-4 diffractometer	*R*_int_ = 0.047
Radiation source: fine-focus sealed tube	θ_max_ = 67.0°, θ_min_ = 4.0°
graphite	*h* = 0→7
ω/2θ scans	*k* = −26→4
2732 measured reflections	*l* = −10→10
2075 independent reflections	3 standard reflections every 120 min
1755 reflections with *I* > 2σ(*I*)	intensity decay: 1.0%

### Refinement

**Table:**

Refinement on *F*^2^	Secondary atom site location: difference Fourier map
Least-squares matrix: full	Hydrogen site location: inferred from neighbouring sites
*R*[*F*^2^ > 2σ(*F*^2^)] = 0.073	H atoms treated by a mixture of independent and constrained refinement
*wR*(*F*^2^) = 0.213	*w* = 1/[σ^2^(*F*_o_^2^) + (0.1492*P*)^2^ + 0.3098*P*] where *P* = (*F*_o_^2^ + 2*F*_c_^2^)/3
*S* = 1.08	(Δ/σ)_max_ < 0.001
2075 reflections	Δρ_max_ = 0.78 e Å^−^^3^
140 parameters	Δρ_min_ = −0.58 e Å^−^^3^
0 restraints	Extinction correction: *SHELXL97* (Sheldrick, 2008), Fc^*^=kFc[1+0.001xFc^2^λ^3^/sin(2θ)]^-1/4^
Primary atom site location: structure-invariant direct methods	Extinction coefficient: 0.029 (4)

### Special details

**Table:**

Geometry. All e.s.d.'s (except the e.s.d. in the dihedral angle between two l.s. planes) are estimated using the full covariance matrix. The cell e.s.d.'s are taken into account individually in the estimation of e.s.d.'s in distances, angles and torsion angles; correlations between e.s.d.'s in cell parameters are only used when they are defined by crystal symmetry. An approximate (isotropic) treatment of cell e.s.d.'s is used for estimating e.s.d.'s involving l.s. planes.
Refinement. Refinement of *F*^2^ against ALL reflections. The weighted *R*-factor *wR* and goodness of fit *S* are based on *F*^2^, conventional *R*-factors *R* are based on *F*, with *F* set to zero for negative *F*^2^. The threshold expression of *F*^2^ > σ(*F*^2^) is used only for calculating *R*-factors(gt) *etc*. and is not relevant to the choice of reflections for refinement. *R*-factors based on *F*^2^ are statistically about twice as large as those based on *F*, and *R*- factors based on ALL data will be even larger.

### Fractional atomic coordinates and isotropic or equivalent isotropic displacement parameters (Å^2^)

**Table:**

	*x*	*y*	*z*	*U*_iso_*/*U*_eq_	
C1	0.4133 (5)	0.36281 (13)	0.1597 (3)	0.0566 (7)	
C2	0.6038 (6)	0.3479 (2)	0.1164 (5)	0.0806 (10)	
H2	0.6706	0.3751	0.0637	0.097*	
C3	0.6932 (8)	0.2901 (3)	0.1549 (7)	0.1103 (19)	
H3	0.8212	0.2782	0.1263	0.132*	
C4	0.5957 (12)	0.2509 (2)	0.2337 (7)	0.117 (2)	
H4	0.6587	0.2128	0.2591	0.141*	
C5	0.4070 (11)	0.26677 (19)	0.2757 (6)	0.1049 (15)	
H5	0.3418	0.2397	0.3297	0.126*	
C6	0.3138 (7)	0.32271 (16)	0.2382 (4)	0.0734 (9)	
H6	0.1838	0.3337	0.2655	0.088*	
C7	0.0475 (5)	0.41022 (15)	−0.1737 (4)	0.0627 (8)	
C8	0.0133 (7)	0.42667 (17)	−0.3429 (4)	0.0721 (9)	
H8	0.0582	0.4689	−0.3459	0.087*	
C9	0.1661 (13)	0.3903 (4)	−0.4061 (7)	0.162 (3)	
H9A	0.1324	0.3484	−0.3988	0.194*	
H9B	0.3216	0.3980	−0.3470	0.194*	
H9C	0.1443	0.4008	−0.5139	0.194*	
C10	−0.2332 (10)	0.4217 (3)	−0.4352 (6)	0.123 (2)	
H10A	−0.3227	0.4479	−0.3908	0.148*	
H10B	−0.2835	0.3809	−0.4321	0.148*	
H10C	−0.2514	0.4332	−0.5420	0.148*	
N1	0.2199 (5)	0.44210 (13)	−0.0704 (3)	0.0632 (8)	
H1N	0.280 (7)	0.4703 (19)	−0.101 (5)	0.076*	
O1	0.1032 (5)	0.43906 (11)	0.1775 (3)	0.0754 (7)	
O2	0.4768 (5)	0.47818 (11)	0.1727 (3)	0.0779 (8)	
O3	−0.0530 (5)	0.37102 (14)	−0.1283 (3)	0.0923 (10)	
S1	0.29657 (13)	0.43493 (3)	0.12050 (8)	0.0570 (4)	

### Atomic displacement parameters (Å^2^)

**Table:**

	*U*^11^	*U*^22^	*U*^33^	*U*^12^	*U*^13^	*U*^23^
C1	0.0624 (17)	0.0556 (16)	0.0489 (15)	−0.0002 (12)	0.0118 (13)	−0.0021 (11)
C2	0.0643 (19)	0.094 (3)	0.084 (2)	−0.0031 (18)	0.0227 (17)	−0.020 (2)
C3	0.074 (3)	0.120 (4)	0.122 (4)	0.030 (3)	0.005 (3)	−0.043 (3)
C4	0.137 (5)	0.080 (3)	0.105 (4)	0.038 (3)	−0.012 (3)	−0.005 (3)
C5	0.144 (4)	0.071 (2)	0.094 (3)	0.014 (3)	0.026 (3)	0.021 (2)
C6	0.090 (2)	0.0648 (19)	0.0673 (19)	0.0021 (17)	0.0261 (17)	0.0103 (15)
C7	0.0627 (17)	0.0669 (18)	0.0547 (17)	−0.0113 (14)	0.0112 (14)	0.0026 (13)
C8	0.083 (2)	0.076 (2)	0.0534 (18)	−0.0139 (17)	0.0147 (16)	−0.0006 (15)
C9	0.184 (7)	0.226 (8)	0.096 (4)	0.083 (6)	0.074 (4)	0.015 (5)
C10	0.113 (4)	0.163 (5)	0.068 (3)	−0.016 (3)	−0.015 (3)	0.003 (3)
N1	0.0731 (17)	0.0619 (16)	0.0497 (14)	−0.0170 (12)	0.0104 (12)	0.0071 (11)
O1	0.0876 (17)	0.0760 (16)	0.0715 (15)	0.0129 (12)	0.0370 (13)	0.0004 (11)
O2	0.0986 (18)	0.0689 (14)	0.0570 (13)	−0.0292 (12)	0.0081 (12)	−0.0041 (10)
O3	0.0977 (19)	0.108 (2)	0.0663 (15)	−0.0501 (16)	0.0162 (13)	0.0048 (13)
S1	0.0701 (6)	0.0519 (5)	0.0477 (5)	−0.0063 (3)	0.0153 (4)	−0.0007 (3)

### Geometric parameters (Å, °)

**Table:**

C1—C2	1.372 (5)	C7—C8	1.507 (4)
C1—C6	1.379 (5)	C8—C9	1.466 (7)
C1—S1	1.747 (3)	C8—C10	1.499 (6)
C2—C3	1.400 (7)	C8—H8	0.9800
C2—H2	0.9300	C9—H9A	0.9600
C3—C4	1.361 (8)	C9—H9B	0.9600
C3—H3	0.9300	C9—H9C	0.9600
C4—C5	1.361 (8)	C10—H10A	0.9600
C4—H4	0.9300	C10—H10B	0.9600
C5—C6	1.367 (6)	C10—H10C	0.9600
C5—H5	0.9300	N1—S1	1.637 (3)
C6—H6	0.9300	N1—H1N	0.81 (4)
C7—O3	1.202 (4)	O1—S1	1.420 (3)
C7—N1	1.378 (4)	O2—S1	1.435 (2)
			
C2—C1—C6	121.7 (3)	C9—C8—H8	107.5
C2—C1—S1	119.7 (3)	C10—C8—H8	107.5
C6—C1—S1	118.6 (3)	C7—C8—H8	107.5
C1—C2—C3	117.1 (4)	C8—C9—H9A	109.5
C1—C2—H2	121.4	C8—C9—H9B	109.5
C3—C2—H2	121.4	H9A—C9—H9B	109.5
C4—C3—C2	121.0 (4)	C8—C9—H9C	109.5
C4—C3—H3	119.5	H9A—C9—H9C	109.5
C2—C3—H3	119.5	H9B—C9—H9C	109.5
C5—C4—C3	120.8 (4)	C8—C10—H10A	109.5
C5—C4—H4	119.6	C8—C10—H10B	109.5
C3—C4—H4	119.6	H10A—C10—H10B	109.5
C4—C5—C6	119.7 (5)	C8—C10—H10C	109.5
C4—C5—H5	120.1	H10A—C10—H10C	109.5
C6—C5—H5	120.1	H10B—C10—H10C	109.5
C5—C6—C1	119.7 (4)	C7—N1—S1	125.3 (2)
C5—C6—H6	120.1	C7—N1—H1N	120 (3)
C1—C6—H6	120.1	S1—N1—H1N	114 (3)
O3—C7—N1	120.9 (3)	O1—S1—O2	118.90 (16)
O3—C7—C8	125.3 (3)	O1—S1—N1	110.43 (16)
N1—C7—C8	113.7 (3)	O2—S1—N1	103.49 (14)
C9—C8—C10	113.7 (5)	O1—S1—C1	108.82 (15)
C9—C8—C7	109.5 (4)	O2—S1—C1	108.49 (16)
C10—C8—C7	110.9 (4)	N1—S1—C1	105.91 (14)
			
C6—C1—C2—C3	0.2 (5)	O3—C7—N1—S1	−4.4 (5)
S1—C1—C2—C3	178.2 (3)	C8—C7—N1—S1	179.4 (3)
C1—C2—C3—C4	−0.8 (7)	C7—N1—S1—O1	−50.6 (3)
C2—C3—C4—C5	0.6 (8)	C7—N1—S1—O2	−178.9 (3)
C3—C4—C5—C6	0.2 (8)	C7—N1—S1—C1	67.1 (3)
C4—C5—C6—C1	−0.8 (7)	C2—C1—S1—O1	−179.6 (3)
C2—C1—C6—C5	0.6 (6)	C6—C1—S1—O1	−1.4 (3)
S1—C1—C6—C5	−177.5 (3)	C2—C1—S1—O2	−48.8 (3)
O3—C7—C8—C9	−88.9 (6)	C6—C1—S1—O2	129.3 (3)
N1—C7—C8—C9	87.0 (5)	C2—C1—S1—N1	61.7 (3)
O3—C7—C8—C10	37.3 (6)	C6—C1—S1—N1	−120.1 (3)
N1—C7—C8—C10	−146.7 (4)		

### Hydrogen-bond geometry (Å, °)

**Table:**

*D*—H···*A*	*D*—H	H···*A*	*D*···*A*	*D*—H···*A*
N1—H1N···O2^i^	0.81 (4)	2.12 (5)	2.898 (4)	160 (4)

Symmetry codes: (i) −*x*+1, −*y*+1, −*z*.
